# CRISPR-Cas Systems and the Paradox of Self-Targeting Spacers

**DOI:** 10.3389/fmicb.2019.03078

**Published:** 2020-01-22

**Authors:** Franziska Wimmer, Chase L. Beisel

**Affiliations:** ^1^Helmholtz Institute for RNA-Based Infection Research (HIRI), Helmholtz Centre for Infection Research (HZI), Würzburg, Germany; ^2^Medical Faculty, University of Würzburg, Würzburg, Germany

**Keywords:** CRISPR-Cas, spacer acquisition, anti-CRISPR proteins, autoimmunity, gene regulation

## Abstract

CRISPR-Cas immune systems in bacteria and archaea record prior infections as spacers within each system’s CRISPR arrays. Spacers are normally derived from invasive genetic material and direct the immune system to complementary targets as part of future infections. However, not all spacers appear to be derived from foreign genetic material and instead can originate from the host genome. Their presence poses a paradox, as self-targeting spacers would be expected to induce an autoimmune response and cell death. In this review, we discuss the known frequency of self-targeting spacers in natural CRISPR-Cas systems, how these spacers can be incorporated into CRISPR arrays, and how the host can evade lethal attack. We also discuss how self-targeting spacers can become the basis for alternative functions performed by CRISPR-Cas systems that extend beyond adaptive immunity. Overall, the acquisition of genome-targeting spacers poses a substantial risk but can aid in the host’s evolution and potentially lead to or support new functionalities.

## Introduction

CRISPR-Cas systems represent highly diverse adaptive immune systems found in many bacteria and most archaea (Barrangou et al., 2007; Sorek et al., 2013; Koonin et al., 2017). These systems consist of two general parts: Clustered Regularly Interspaced Short Palindromic Repeats (CRISPR) arrays and CRISPR-associated (Cas) proteins. CRISPR arrays represent the immunological memory of prior infections encoded within individual spacers separated by conserved repeats. Cas proteins carry out the adaptive immune functions. The Cas proteins are highly diverse, resulting in CRISPR-Cas systems currently being grouped into two classes, six types, and over 30 subtypes (Makarova et al., 2015; Koonin et al., 2017; Koonin and Makarova, 2019).

While the specific proteins and biomolecular mechanisms vary, all systems act through three general steps as part of adaptive immunity. The first step, acquisition, incorporates pieces of invading nucleic acids, called protospacers, as new spacers within the CRISPR array. The protospacers are often selected based on the presence of a flanking protospacer adjacent motif (PAM) (Yosef et al., 2013; Wang et al., 2015). Acquisition requires the universal Cas proteins Cas1 and Cas2 (Yosef et al., 2012; Nuñez et al., 2014), although other accessory factors such as Cas4 (Kieper et al., 2018), Csa1 (Liu T. et al., 2017), Csn2 (Heler et al., 2015; Wei et al., 2015) and reverse transcriptase (RT) (Kojima and Kanehisa, 2008; Simon and Zimmerly, 2008; Silas et al., 2016) can also be involved. In type II CRISPR-Cas systems, the effector nuclease Cas9 can also play an essential role in the acquisition of new spacers (Heler et al., 2015; Wei et al., 2015). The acquired spacers serve as DNA records of prior infections that are passed to the host’s progeny.

The second and third steps involve the biogenesis of CRISPR RNAs (crRNAs) from the CRISPR arrays followed by crRNA-directed immune defense. As part of crRNA biogenesis, the CRISPR array is, for most cases, transcribed into a long precursor CRISPR RNA (pre-crRNA) and processed into mature crRNAs by Cas proteins. In some cases, processing involves accessory factors such as RNase III (Carte et al., 2008; Deltcheva et al., 2011; Behler et al., 2018; Lee et al., 2018, 2019). The crRNA then forms a complex with Cas effector proteins to target foreign nucleic acids. Class 2 CRISPR-Cas systems rely on only one protein to bind and cleave their targets, with type II systems and some type V systems also requiring a *trans-*activating crRNA (tracrRNA) for effector complex formation (Deltcheva et al., 2011; Shmakov et al., 2015; Zetsche et al., 2015). Class I systems in contrast rely on multiple proteins that form a multi-subunit effector complex (Brouns et al., 2008; Hale et al., 2009). The resulting ribonucleoprotein complex then surveils the host’s cytoplasm for DNA and/or RNA sequences that are complementary to the spacer and flanked either by a PAM or a sequence lacking complementarity to the corresponding portion of the crRNA repeat (Mojica et al., 2005; Marraffini and Sontheimer, 2010; Leenay and Beisel, 2017; Meeske and Marraffini, 2018).

One commonality across CRISPR-Cas systems is their reliance on the array-encoded spacers to direct CRISPR-based immunity. To date, only 1–19% of identified spacers have been matched to potential protospacer sites, where most of the assigned spacers appear to be derived from the genome of bacteriophages (herein called phages), archaeal viruses (herein called viruses), plasmids or other organisms (Bolotin et al., 2005; Mojica et al., 2005; Pourcel et al., 2005; Marraffini and Sontheimer, 2008; Brodt et al., 2011; Bikard et al., 2012; Shmakov et al., 2017). However, many of the assigned spacers match sequences within the host genome, what are generally called self-targeting spacers.

**FIGURE 1:**
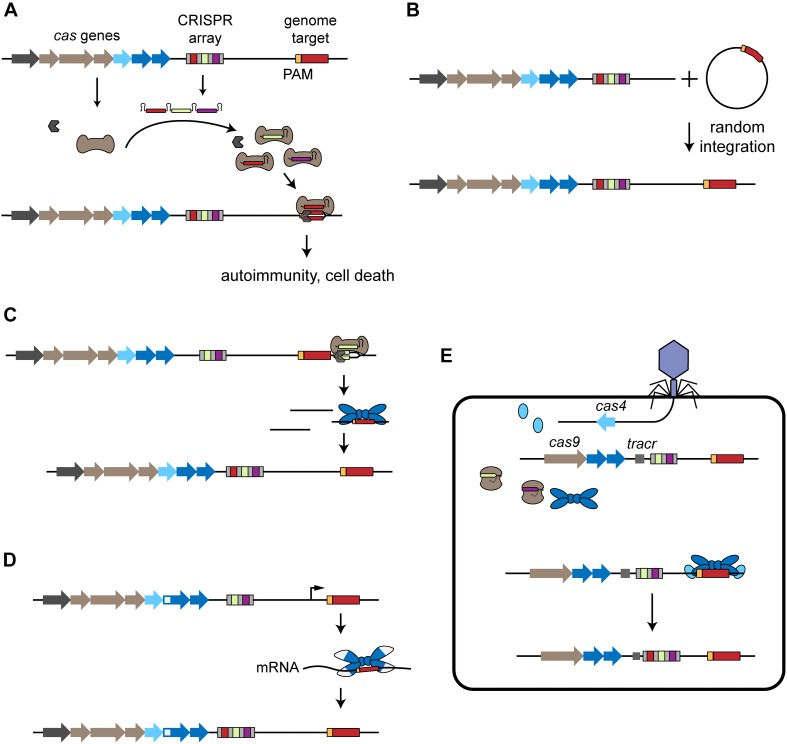
Acquisition of self-targeting spacers. **(A)** Overview of self-targeting by CRISPR-Cas systems. The CRISPR array is transcribed and processed into individual crRNAs that form a ribonucleoprotein complex with the Cas effector proteins (brown). One of the crRNAs encodes a self-targeting spacer (red) that directs binding to the complementary protospacer sequence (red) flanked by a PAM (orange) located on the genome, leading to autoimmunity and cell death. **(B)** Mobile genetic elements harboring a CRISPR-Cas target sequence can be incorporated into the host chromosome, leading to self-targeting. **(C)** Primed acquisition. The CRISPR effector complex recognizes a target, potentially generating cleaved products. These products can then be incorporated into the CRISPR array by the acquisition complex (blue), leading to acquisition of self-targeting spacers. **(D)** Spacer acquisition from RNA. RT-Cas1 forms a complex with Cas2 (white and blue) and leads to incorporation of self-targeting spacers derived from the host’s RNA. **(E)** Virally driven acquisition of self-targeting spacers. The phage injects its genome into the host cell and the encoded *cas4* is expressed. In cooperation with the host’s endogenous acquisition complex, the phage-derived Cas4 leads to the incorporation of genome-derived spacers into the host’s CRISPR array.

Self-targeting spacers are unexpected due to an observed preference toward acquiring foreign genetic material (Levy et al., 2015) and heavy cytotoxicity to the host because self-targeting of the host’s chromosome would lead to cell death (Stern et al., 2010; Jiang et al., 2013; Vercoe et al., 2013; Gomaa et al., 2014; Figure 1A). Here, we review the presence and consequences of self-targeting spacers. We address the known distribution of self-targeting spacers in sequenced CRISPR-Cas systems. We then discuss different mechanisms of acquisition that could generate self-targeting spacers and how these organisms can survive despite the potential for chromosomal targeting and autoimmunity. Finally, we report some of the beneficial functions that have been associated with the self-targeting spacers that can imbue CRISPR-Cas systems with functionalities that extend beyond adaptive immunity. This content greatly expands on an earlier mini-review on the consequences of chromosomal targeting (Heussler and O’Toole, 2016) and incorporates recently reported examples of self-targeting reflecting alternative functions of these prevalent adaptive immune systems.

## Natural Occurrence of Self-Targeting Spacers

Multiple studies have explored the source of spacers in diverse CRISPR-Cas systems, with recurring observations of self-targeting spacers. In the first broad study of matching protospacers, 88 of the analyzed 4,500 spacers were similar to known sequences, and 35% of these spacers matched chromosomal DNA not directly related to foreign genetic elements (Mojica et al., 2005). Separately, a study from 2008 found that 7% of spacers in different CRISPR-Cas systems from *Streptococcus thermophilus* matched chromosomal sequences (Horvath et al., 2008). One year later, the same group analyzed CRISPR-Cas systems from a more diverse set of lactic acid bacteria, reporting that 23 of the 104 spacers matched the chromosome (Horvath et al., 2009). Shortly thereafter, one study analyzed the CRISPR arrays of the 330 prokaryotes containing CRISPR-Cas systems included in the CRISPRdb database (Grissa et al., 2007) in 2010, with self-targeting spacers comprising 0.4% of all spacers (including the vast majority of spacers with no assignable protospacers) and appearing in 18% of the included prokaryotic genomes (Stern et al., 2010).

The number of sequenced organisms has increased over time, allowing more recent studies to more deeply and widely interrogate spacer origins. For instance, one study in 2017 screened ∼50,000 completely or partially assembled genomes, while another study in 2018 used the online tool CRISPRminer to evaluate more than 60,000 organisms harboring a CRISPR array (Shmakov et al., 2017; Zhang et al., 2018). Shmakov et al. assigned protospacer locations to 7% of the detected 363,460 unique spacers, with ∼6% of these spacers matching prokaryotic genomes and 16% of these genome-matching spacers being potentially unrelated to (pro-)viral sequences (Shmakov et al., 2017). The study with CRISPRminer reported 22,110 self-targeting events in publications (Stern et al., 2010; Rauch et al., 2017; Watters et al., 2018) and could predict 6,260 additional putative self-targeting spacers in 4,136 organisms, implying that ∼7% of the genomes within their database should harbor at least one self-targeting spacer (Zhang et al., 2018).

The natural acquisition of self-targeting spacers has also been observed as part of adaptive evolution studies between phages and their prokaryotic host. Two key studies relied on a strain of the bacterium *S. thermophilus* harboring two type II-A CRISPR-Cas systems (Paez-Espino et al., 2013, 2015). In these studies, only 0.01 – 0.04% of the observed new spacers matched the genome. These frequencies are lower than those reported in the large-scale bioinformatics studies, although this discrepancy can be attributed in part to the selective pressure exerted by the actively infecting phages.

## Incorporation of Self-Targeting Spacers

Given the frequency of self-targeting spacers and their potential for autoimmunity, we next discuss the circumstances under which a self-targeting spacer can be acquired. In particular, we consider five general scenarios that have been reported: naïve acquisition from DNA, protospacers within a transferred mobile genetic element (MGE), primed adaptation, naïve acquisition from RNA, and phage/virus-triggered acquisition from host DNA. For many of these scenarios, we address the extent to which acquisition differentiates between chromosomal and foreign genetic material and the known associated mechanisms. We finally must note that our understanding of CRISPR-based acquisition is still developing, and other mechanisms within the diversity of CRISPR-Cas systems likely await discovery.

### Naïve Acquisition

Naïve acquisition leads to the incorporation of new spacers without any influence from the existing pool of spacers. Cas1 and Cas2 are required while Cas4, Csn2 or Cas9 may be additionally needed depending on the system sub-type (Yosef et al., 2012; Nuñez et al., 2014; Heler et al., 2015; Wei et al., 2015; Kieper et al., 2018). It was known for many years that protospacers were commonly flanked by PAMs to allow targeting by the effector proteins to differentiate between self and non-self targets (Deveau et al., 2008; Horvath et al., 2008). However, it remained unclear how the acquisition machinery differentiated between invader and chromosomal DNA. In one of the first studies to systematically interrogate spacer acquisition, Levy and coworkers sequenced over 38 million newly acquired spacers following plasmid-based expression of Cas1 and Cas2 in an *Escherichia coli* strain harboring a CRISPR array but lacking endogenous *cas* genes. They found that spacers were preferentially acquired from replication forks, presumably due to stalling during replication and degradation by RecBCD. This preference resulted in 100-fold to 1,000-fold enrichment of spacers derived from a resident plasmid compared to the chromosome. The high-copy number plasmids present most of the replication forks in a replicating cell, partly explaining the preference toward high copy plasmids (Levy et al., 2015).

Another critical factor identified by Levy and coworkers was the presence of Chi sites. These sequence motifs interact with and prevent DNA degradation by RecBCD (Smith, 2012) at the sites of double-stranded DNA breaks that often occur at stalled replication forks (Kuzminov, 2001; Michel et al., 2001). Due to the fact that Chi sites occur approximately every 5 kb in the *E. coli* genome (El Karoui et al., 1999), these Chi sites were hypothesized to mark the host DNA as “self” and prevent acquisition of spacers from the host’s genome. The plasmids contained fewer Chi sites, likely further contributing to preferential acquisition from this DNA. Linear viral DNA would also offer a preferred substrate for RecBCD, resulting in DNA fragments that can be used to generate new spacers (Levy et al., 2015). Phages are known to encode RecBCD inhibitors and some also encode a large number of Chi sites in their genome (Friedman and Hays, 1986; Murphy and Lewis, 1993; Bobay et al., 2013), thus potentially countering acquisition by CRISPR-Cas systems.

Liu et al. observed a different element influencing naïve acquisition in *Sulfolobus islandicus*, an archaeon that encodes one type I-A CRISPR-Cas system and two type III-B CRISPR-Cas systems. Following overexpression of Csa3a that drives expression of the type I-A acquisition genes, *S. islandicus* integrated spacers from the *csa3a* expression plasmid as well as from its own genome with a high bias toward the plasmid (Liu et al., 2015). Interestingly, for deletion mutants lacking RNA processing or nuclease activity, <28% of spacers were derived from the plasmid (Liu T. et al., 2017). While this fraction was far less than the >90% in a previous study (Liu et al., 2015), it still reflected preferential acquisition from plasmids when taking into account the relative length of the plasmid and chromosomal DNA (Liu T. et al., 2017). The stronger preference for plasmid DNA in the presence of an active CRISPR-Cas system may be explained in part by the cytotoxicity of genome targeting by the active but not impaired system upon self-targeting.

Spacer acquisition in type II CRISPR-Cas systems also appears to differ for active versus impaired CRISPR-Cas systems. Wei et al. (2015) looked at acquisition requirements in a type II-A CRISPR-Cas system by expressing the different CRISPR-Cas components on plasmids and monitoring spacer acquisition. They found that acquisition required the presence of Cas9, in contrast to spacer acquisition by Cas1 and Cas2 in type I CRISPR-Cas systems. Interestingly, the authors found that the cleavage activity of Cas9 contributed to an observed preference for acquisition from plasmid DNA. Specifically, by using a mutated Cas9 that disrupts its cleavage activity (dCas9), the authors shifted the fraction of plasmid-derived spacers from 68% to 4%, representing a loss of preference given the matching ratio of plasmid DNA to genomic DNA (Wei et al., 2015). In total, naïve acquisition by different types of CRISPR-Cas systems can lead to the incorporation of self-targeting spacers, although foreign genetic material is the predominant source of spacers. It would be interesting to investigate if the above reported phenomena can also be observed in different organisms or other CRISPR-Cas systems that rely on additional Cas proteins for acquiring new spacers.

### Protospacers Within Transferred Mobile Genetic Elements

Many self-targeting spacers identified in nature bear homology to MGEs such as transposons or prophages/proviruses that have been incorporated into the genome. These spacers could have been acquired prior to the incorporation of the MGE as a preventative measure, or afterward to induce cell death and prevent further spread of the MGE (Figure 1B). All evidence of this mechanism comes from bioinformatic experiments. Looking at self-targeting spacers with 100% complementary to their predicted protospacer region, Stern et al. (2010) found an approximately equal distribution of protospacers from mobile elements encoded in the chromosome and non-mobile elements (47% vs. 53%). In comparison, Shmakov et al. (2017) assigned 83% of the self-targeting spacers to (pro-)phage sequences. The difference might arise from the greater abundance of sequenced MGEs over time (Geer et al., 2010; Akhter et al., 2012). Nevertheless, these frequencies leave ample spacers derived from non-mobile elements.

### Primed Adaptation

A different potential means of incorporating self-targeting spacers is through primed adaptation (or primed acquisition). Acquisition of spacers under primed adaptation requires target recognition with pre-existing spacers that are partially or fully complementary to the foreign DNA. Recognition leads to the acquisition of multiple spacers from sites in close proximity to the existing protospacer (Datsenko et al., 2012; Swarts et al., 2012; Richter et al., 2014; Jackson et al., 2019; Figure 1C).

Bioinformatic evidence indicates that primed adaptation is widespread in type I and type II CRISPR-Cas systems (Nicholson et al., 2019). Primed adaptation by type I systems involves degradation of the target site by Cas3 and incorporation of the degradation products as new spacers by Cas1 and Cas2 (Künne et al., 2016). Primed adaptation by type II systems is not well understood, although Nicholson et al. (2019) proposed two possible pathways: one that involves a main role of Cas9, and another involving host-specific processes such as DNA repair producing pre-spacers at the sites of target cleavage.

Regardless of the exact mechanism, primed adaptation is expected to preferentially incorporate foreign genetic material due to the pre-existence of more spacers derived from non-chromosomal elements. However, primed acquisition of host DNA could occur upon targeting MGE that were incorporated into a bacterial or archaeal genome (Nicholson et al., 2019). Primed acquisition outside of the borders of the MGE could also be triggered, leading to incorporation of non-mobile self DNA from the chromosome. Finally, spacers that evolved to target foreign DNA might prime with similar sequences in chromosomal DNA (Staals et al., 2016), where prior work showed that priming can occur even with 13 mutations in the target site relative to the pre-existing spacer (Fineran et al., 2014).

### Naïve Acquisition of RNA-Derived Spacers

One unique mode of acquisition is through relatively rare Cas proteins that recognize RNA rather than DNA. These proteins include a RT often translationally fused to Cas1 or to a fusion between Cas1 and the Cas6 protein responsible for crRNA biogenesis. This unique RNA-acquiring machinery is predominantly associated with type III CRISPR-Cas systems but is also found with type I-E and type VI-A systems (Kojima and Kanehisa, 2008; Simon and Zimmerly, 2008; Toro and Nisa-Martínez, 2014; Silas et al., 2017; Toro et al., 2019a, b). For the few examples that have been studied, these RTs reverse-transcribe an acquired RNA into DNA to produce a substrate for acquisition (Silas et al., 2016). If the RNA-derived spacers are derived from host RNA, the associated type III CRISPR-Cas systems can now target the host and lead to autoimmunity (Figure 1D). Interestingly, self-targeting spacers have been found in three strains encoding a RT as part of their type III CRISPR-Cas systems (Silas et al., 2017; Zhang et al., 2018).

Other systems solely encode RT and Cas1 and lack all other Cas proteins, holding the potential to acquire self-targeting spacers without inducing autoimmunity. As one example, *Rivularia* sp. PCC 7116 encodes Cas1, Cas2, and RT in a distinct genomic island compared to the other CRISPR-Cas systems present in that bacterium. The CRISPR array associated with Cas1, Cas2 and RT harbors a spacer matching a hypothetical gene encoded on the bacterial chromosome (Silas et al., 2017; Kersey et al., 2018; Zhang et al., 2018). The lack of effector proteins suggests that these systems are used for alternative functions rather than immunity, although this has not been investigated to-date.

The unique sourcing of spacers from RNA raises questions about how the acquisition machinery selected some RNA sequences over others. Silas and coworkers sequenced the spacer content in an open-air culture of *Arthrospira platensis*, which encodes a RT-Cas1 fusion as part of its type III-B CRISPR-Cas system (Silas et al., 2017). Most of the associated protospacers could not be identified, and the few that could be identified traced to DNA viruses. Schmidt and coworkers were able to gain more extensive insights by monitoring spacer acquisition in *E. coli* following plasmid-based expression of the type III *Fusicatenibacter saccharivorans* RT-Cas1 and Cas2. While spacers were derived from RNAs encoded in the chromosome and plasmid, there was a strong preference for A/T-rich sequences at the ends of highly expressed genes. Interestingly, there was no obvious preference for a flanking motif or for plasmid-encoded RNAs (Schmidt et al., 2018). Further studies are needed to fully understand preferences exhibited by type III CRISPR-Cas systems for RNA acquisition.

### Acquisition of Self-Targeting Spacers Triggered by Foreign Invaders

There is also evidence that phages can encode Cas proteins that drive endogenous CRISPR-Cas systems to preferentially acquire self-targeting spacers. The first direct evidence comes from studying the origin of spacers encoded within the CRISPR array of *Campylobacter jejuni* PT14 harboring a minimal type II-C CRISPR-Cas system (Hooton and Connerton, 2014). While the spacers do not share 100% sequence identity with any known sequences, some of the spacers partially matched chromosomal sequences in the PT14 genome. Tracking spacer content in a co-culture of PT14 cells and CP8/CP30A phage revealed that all newly acquired spacers were derived from the host’s chromosome and not the phage (Hooton and Connerton, 2014). The phage encoded a copy of the *cas4* gene involved in protospacer maturation as part of many CRISPR-Cas systems (Zhang et al., 2012; Lemak et al., 2013; Kieper et al., 2018; Lee et al., 2018), while the endogenous type II-C CRISPR-Cas system normally lacks this gene. The authors therefore attributed the unexpected self-targeting acquisition events to the phage encoded Cas4 (Hooton and Connerton, 2014; Figure 1E).

Further evidence that viral Cas4 can impact host acquisition was found in *S. islandicus.* Zhang and coworkers evaluated the impact of the viral *cas4* gene found in a *Sulfolobus* spindle-shaped virus by transforming a plasmid encoding the viral *cas4* into *S. islandicus*. Cells harboring the plasmid exhibited less frequent spacer acquisition, although the frequency of spacers acquired from the plasmid or chromosome did not change. Furthermore, overexpression of host Cas4 from a plasmid also led to reduced spacer acquisition. These findings suggest that overproduction of Cas4 can in some cases disable spacer acquisition. One explanation is that the viral encoded Cas4 serves as an anti-CRISPR protein (Acr) by preventing spacer acquisition and in turn enabling escape from CRISPR-Cas targeting (Zhang et al., 2019). While more work is needed to elucidate the underlying role of the virally encoded Cas4, these examples and the many other instances of virally encoded Cas4 (Krupovic et al., 2015; Hudaiberdiev et al., 2017) suggest the intriguing possibility that phages and viruses could be actively directing the acquisition of spacers.

## Surviving Self-Targeting by CRISPR-Cas Systems

Unrelated to how prokaryotes incorporate spacers that target their own genome, cells must overcome self-targeting by their own CRISPR-Cas system to survive. CRISPR-based interference against the host’s own genome is expected to lead to lethal autoimmunity due to the nuclease cutting within or close to their target site. Repair mechanisms in prokaryotes are often not efficient enough to fix CRISPR-Cas induced DNA damage, and DNA breaks often result in cell death (Stern et al., 2010; Jiang et al., 2013; Vercoe et al., 2013; Gomaa et al., 2014). Nevertheless, many different examples exist in which a self-targeting spacer can be tolerated. Below we describe each known mechanism.

### Active DNA Repair

**FIGURE 2:**
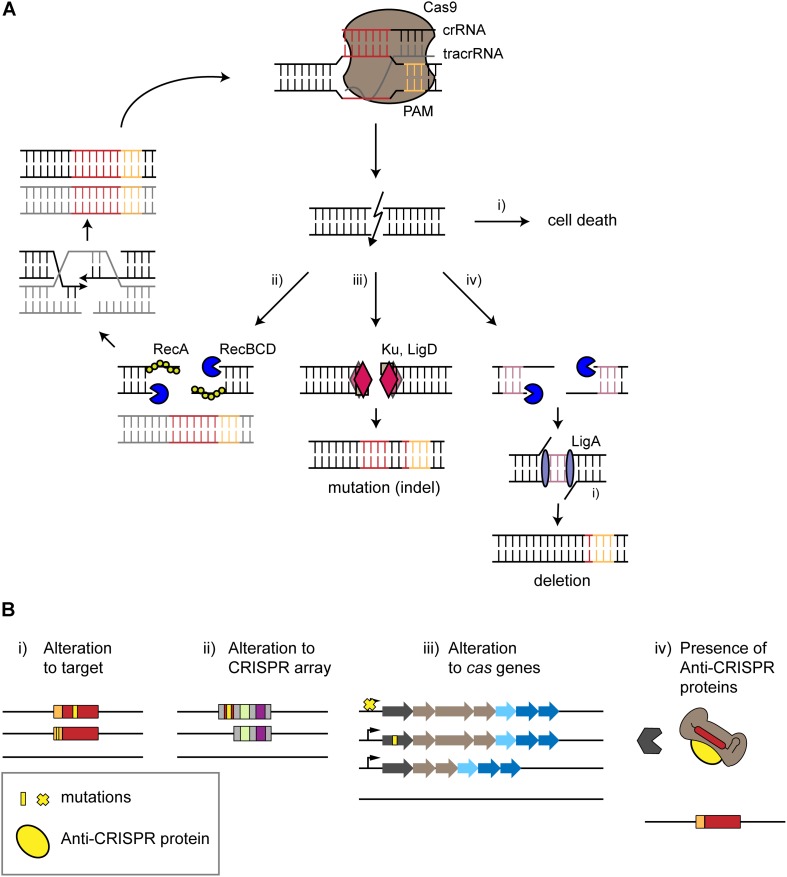
Surviving self-targeting. **(A)** Survival of CRISPR-Cas targeting by intrinsic repair mechanisms. The type II effector protein Cas9 is used as an example. The CRISPR effector complex binds to its target in the genome (red) next to a PAM (orange), leading to a double-stranded break (DSB) causing different outcomes. **(i)** Cell death occurs if the break is not repaired. **(ii)** Homology-directed repair (HDR) restores the target site in the presence of an intact copy of the chromosome. DNA ends of the DSB undergo trimming in a Rec-dependent manner. HDR leads to the restoration of a chromosome that undergoes further attack by the CRISPR-Cas system. **(iii)** Non-homologous end joining leads to the formation of an insertion or deletion (indel). End joining is mediated by the repair proteins Ku and LigD. **(iv)** Alternative end-joining leads to deletions. DNA ends are trimmed by RecBCD until micro-homologous regions (purple) are reached. These regions are then ligated by LigA, resulting in deletions. **(B)** Escape from autoimmunity through mutations, deletions or active inhibition. **(i,ii)** mutations (yellow) or deletions within the protospacer, PAM or CRISPR array disrupts self-targeting. **(iii)** Mutation of the *cas* operon, inhibition of Cas expression or deletion of a *cas* gene or the entire locus can also prevent self-targeting. **(iv)** Anti-CRISPR proteins encoded within an integrated prophage can block CRISPR-Cas interference through different mechanisms, such as binding the Cas effector protein to prevent PAM recognition.

CRISPR-based targeting would be expected to induce irreparable damage, lest the cells repair invading genetic material and allow an infection to persist. Accordingly, many studies have reported that chromosomal targeting by CRISPR nucleases is cytotoxic in different bacteria and archaea (Stern et al., 2010; Jiang et al., 2013; Vercoe et al., 2013; Bikard et al., 2014; Citorik et al., 2014; Gomaa et al., 2014; Li Y. et al., 2016). That said, there exist examples in which intrinsic DNA repair mechanisms such as homology-directed repair (HDR), non-homologous end joining (NHEJ), and alternative end-joining (A-EJ) mechanisms allow cell survival (Chayot et al., 2010; Tong et al., 2015; Cui and Bikard, 2016; Stachler et al., 2017; Figure 2A).

One potential mechanism is HDR through an additional copy of the chromosome. Cui and Bikard first observed this phenomenon when evaluating the consequences of targeting the *E. coli* chromosome with heterologously expressed Cas9 (Cui and Bikard, 2016). They found that targeting different sites within non-essential genes resulted in RecA-mediated HDR. Targeting did induce the SOS DNA-damage response, although the cells maintained their viability. In a separate example, Stachler and coworkers reported that the archaeon *Haloferax volcanii* could tolerate chromosomal targeting through its endogenous type I-B CRISPR-Cas system (Stachler et al., 2017). However, the tolerance could be attributed in part to the endogenous CRISPR array providing most of the crRNAs in the effector complexes. Deleting the Cas6 processing protein and expressing a mature self-targeting crRNA resulted in a fitness defect that was strengthened by expressing the crRNA at higher levels. The extent of self-targeting in the presence of the endogenous CRISPR array therefore was sufficiently weak to allow repair through HDR and the roughly 20 copies of the *H. volcanii* genome (Zerulla et al., 2014; Stachler et al., 2017). In both of these examples, there would likely be some selective pressure to disrupt self-targeting given the need for continuous repair.

Non-homologous end joining and alternative end-joining offer distinct repair mechanisms that permanently alter the target site, preventing further attack by CRISPR-Cas systems. NHEJ does not utilize a repair template and instead repairs double-stranded DNA breaks (DSBs) by adding insertions or deletions (indels) to the site of the DSB. Some prokaryotes possess relatively unsophisticated NHEJ machinery compared to eukaryotes, typically comprised of the complexes Ku and LigD (Aravind and Koonin, 2001; Weller et al., 2002; Gong et al., 2005; Bowater and Doherty, 2006; Shuman and Glickman, 2007; Tong et al., 2015). Some bacteria such as *E. coli* lacking Ku and LigD can utilize phage ligases to mediate NHEJ-like repair of CRISPR-Cas induced DSBs (Su et al., 2019). While NHEJ efficiently repairs DNA cleaved by some CRISPR nucleases in eukaryotic cells, some CRISPR-Cas induced DNA damage in prokaryotes is still highly cytotoxic when NHEJ is active (Xu et al., 2015; Bernheim et al., 2017). A-EJ is a repair mechanism that relies on microhomology-mediated end joining and largely leads to deletions. DSBs induce extensive end-resection that is mostly dependent on RecBCD, while Ligase-A repairs the break by joining micro-homologous regions of 1 to 9 bps (Chayot et al., 2010; Figure 2A). Prior work suggested that A-EJ led to large deletions following genomic attack by a type I-F CRISPR-Cas system in *Pectobacterium atrosepticum* (Vercoe et al., 2013). Whether repair occurs through NHEJ or A-EJ, the resulting genome would be less susceptible (or even completely unsusceptible) to follow-up attack through the self-targeting spacer.

### Mutations Disrupting CRISPR-Based Targeting

Mutations can prevent efficient CRISPR targeting in multiple ways. One way is mutation of the target site such as through NHEJ or A-EJ, impacting spacer complementarity or PAM recognition (Figure 2B). Two studies evaluating self-targeting through type II-A systems in *S. thermophilus* reported not only mutations of the targeted *lacZ* gene but also deletion of the gene (Selle et al., 2015; Cañez et al., 2019). In one of the studies assessing self-targeting in the *S. thermophilus* strain LMD-9, targeting resulted in loss of ∼1.2 kb that included the *lacZ* gene. These deletions appeared to arise via genomic island excision via recombination between two flanking insertion-sequence elements (Selle et al., 2015) that occur quite frequently in *S. thermophilus* (Bolotin et al., 2004). In contrast, another study reported an ∼40-kb deletion upon targeting *lacZ* in the *S. thermophilus* strain DGCC7710 that shares 99.2% sequence homology to LMD-9. No insertion-sequence elements could be detected within 50 kb flanking the *lacZ* gene, potentially explaining why the same escape mechanism observed in LMD-9 did not take place in DGCC7710. Recombination here might have happened between two regions encoding two *galE* genes sharing 86% nucleotide identity located 3 kb upstream and 30 kb downstream of *lacZ* (Cañez et al., 2019). One interesting possibility is that these large deletions existed in a small fraction of the cell population, where CRISPR-based targeting allowed this sub-population to survive (Selle et al., 2015; Cañez et al., 2019). The different outcomes of self-targeting in *S. thermophilus* LMD-9 and DGCC7710 highlight the different escape mechanisms that can occur even between strains of the same species.

Escape from lethal self-targeting can not only occur via target mutation but also via mutations or deletions within the CRISPR array or the *cas* genes (Figure 2B). In the same study noted above (Cañez et al., 2019), the authors also investigated the escape mechanism by targeting *lacZ* with the endogenous type I-E CRISPR-Cas system in *S. thermophilus* DGCC7710. Surprisingly, no deletions in the target site could be observed, and escape mutants consistently harbored defective plasmids missing the targeting spacer and one repeat likely caused by recombination between repeats that eliminated the self-targeting spacer (Cañez et al., 2019; Figure 2B). Thus, escape mechanisms can differ not only between strains but also between CRISPR-Cas systems. Loss of the plasmid-encoded spacer also occurred as the principal mode of escape when targeting *E. coli*’s genome with its endogenous type I-E system (Gomaa et al., 2014). Separately, as an example of disrupting *cas* genes, *Lactobacillus acidophilus* NCFM appears to have deleted its entire *cas* gene cassette to avoid lethal self-targeting by six genome-targeting spacers encoded in the CRISPR array (Stern et al., 2010; Kersey et al., 2018; Zhang et al., 2018). Furthermore, mutations in the *cas* genes were reported when targeting the staphylococcal cassette chromosome *mec* (SSC*mec*) in *Staphylococcus aureus* through the endogenous III-A CRISPR-Cas system (Guan et al., 2017) or when targeting different sites through the I-F CRISPR-Cas system native to *P. atrosepticum* (Dy et al., 2013). Between disruption of the spacer or *cas* genes, explicit loss of an endogenous self-targeting spacer has been less reported in natural systems. However, this can be explained by bioinformatic searches having difficulties detecting loss of self-targeting spacers in genome databases given that the rest of the CRISPR array may still be intact.

Self-targeting by a CRISPR-Cas system also does not need to drive only one mode of escape. For instance, in the example of self-targeting through the type III-A CRISPR-Cas system in *S. aureus*, the authors reported different mutations or deletions in the escape mutants (Guan et al., 2017). Large deletions that included the target site occurred in ∼90% of the escape mutants, while spacer mutations or loss-of-function mutations in *cas* genes were also detected. Separately, in the example of self-targeting through the type I-F CRISPR-Cas system in *P. atrosepticum*, the bacterium harbors one naturally occurring self-targeting spacer that is not cytotoxic due to a mutation in the target’s PAM (Dy et al., 2013). Transformation of plasmids harboring other self-targeting spacers further led to different sized deletions of regions containing the protospacer or removal of the *cas* operon. The frequency of one escape mode over another likely depends on different factors such as the frequency of background mutation and recombination, the types of mutations that can form, and the fitness defect that they introduce.

### Partially Complementary Spacers Directing Target Binding but Not Cleavage

Mutations to the target site or the spacer can result in partial complementarity between spacers and their protospacers. For some systems, partial complementarity eliminates target cleavage but can preserve target binding. Comparison between off-target binding by dCas9 and off-target cleavage by Cas9 demonstrated extensive off-target binding but not cleavage (Wu et al., 2014). Another study also showed that partial target complementarity could allow an active Cas9 to bind but not cleave DNA, resulting in transcriptional silencing (Bikard et al., 2013). Wu and coworkers proposed a model which would explain the higher specificity of Cas9 by taking binding at the seed region into consideration. They hypothesized that PAM recognition by the Cas9:crRNA complex leads to DNA melting and enables base pairing between the spacer and the complementary seed region. As long as complementarity exists through the seed region, partial base pairing can allow target binding without cleavage (Wu et al., 2014). As a result, organisms could harbor spacers with partial complementarity to their own genome that would still drive target recognition but not autoimmunity.

### RNA Targeting

While we have focused on CRISPR-Cas systems that explicitly target DNA, the type III and VI systems naturally target RNA as part of immune defense (Hale et al., 2009, 2012; Abudayyeh et al., 2016), with distinct implications for self-targeting. Type III CRISPR-Cas systems are capable of targeting DNA and RNA. The system’s Csm or Cmr effector complex is guided to RNA targets complementary to the crRNA, triggering the sequence-specific RNase activity of Csm3 or Cmr4, respectively (Benda et al., 2014; Goldberg et al., 2014; Tamulaitis et al., 2014; Samai et al., 2015). Lack of complementarity between the 5′ crRNA handle and the target RNA activates single-stranded DNase activity by Cas10 (Jung et al., 2015; Kazlauskiene et al., 2016), although there is evidence of a 3′ RNA PAM motif that suggests diverging criteria for target selection across type III systems (Elmore et al., 2016). Furthermore, target recognition by the type III effector complex triggers Cas10 to produce cyclic adenylates. These molecules in turn activate the CRISPR accessory protein Csm6/Csx1, leading to non-specific RNA degradation to assist in viral defense (Kazlauskiene et al., 2017; Niewoehner et al., 2017; Rouillon et al., 2018).

In contrast to type III CRISPR-Cas systems, type VI systems represent the only systems known to-date that exclusively target RNA (Abudayyeh et al., 2016). Cas13, the type VI effector protein, recognizes complementary RNA sequences as long as the repeat-portion of the crRNA cannot extensively base pair with the target (Meeske and Marraffini, 2018). Upon target recognition, Cas13 undergoes a conformational change that activates the effector’s ribonuclease domain, resulting in non-specific cleavage of the proximal portions of the target RNA (Liu et al., 2017a, b). The effector domain remains highly active even after cleavage of local RNAs, leading to the extensive degradation of cellular RNAs. The degradation can be sufficiently extensive to shut down the host’s growth, resulting in a reversible dormancy state (Abudayyeh et al., 2016; Meeske et al., 2019). The activity of type VI CRISPR-Cas targeting also had a more severe effect on the fitness of *E. coli* during high production of target RNA, potentially allowing the cell to survive self-targeting by Cas13 if the target RNA is not highly expressed (Abudayyeh et al., 2016) and sparing the cells from self-targeting induced dormancy.

Another consequence of RNA-based (self-)targeting is type III systems and Cas13 ignoring transcriptionally silent targets. Activation of type III and VI systems only upon RNA recognition would be particularly important for temperate phages and viruses whose lytic genes are repressed during lysogeny (Johnson et al., 1981). Therefore, if a spacer directs the Csm/Cmr effector complex or Cas13 to an RNA necessary for the lytic cycle, then only the lysogens entering the lytic cycle will be targeted. Tolerance of a prophage has been shown for the type III-A system in *Staphylococcus epidermidis* that actively targets its own prophages only upon transition into the lytic cycle (Goldberg et al., 2014). By only targeting phages and viruses in the lytic cycle, cells are able to maintain any potentially positive functions that might arise from a prophage/provirus encoded in their genome and prevent cell death during the invader’s lytic phase.

### Genome-Encoded Anti-CRISPR Proteins

Escape from targeting is not limited to genetically disrupting the CRISPR-Cas system or its target; another means involves inhibiting CRISPR-Cas activity in *trans* by Acrs. These proteins allow phages/viruses to thwart immunity by CRISPR-Cas systems (Pawluk et al., 2018). So far, Acrs have been identified that inhibit different subtypes of type I, II, III, and V CRISPR-Cas systems (Bondy-Denomy et al., 2013; Pawluk et al., 2014, 2016a,b; Hynes et al., 2017; Rauch et al., 2017; He et al., 2018; Marino et al., 2018; Watters et al., 2018; Bhoobalan-Chitty et al., 2019), and Acrs against type IV and VI systems likely await discovery. The Acrs identified to-date have exhibited remarkable diversity in their sequence and in their mechanism of action, such as blocking DNA binding, preventing effector complex formation, sequestering the nuclease into dimers, blocking nuclease activity or preventing nuclease recruitment (Bondy-Denomy et al., 2015; Pawluk et al., 2018; Thavalingam et al., 2019).

Acrs allow phages and viruses to not only escape attack by CRISPR-Cas systems but also protect a lysogenized phage/provirus (not to mention the host chromosome) from an endogenous CRISPR-Cas system encoding a viral-targeting or chromosomal-targeting spacer (Figure 2B). Therefore, a genome encoding both a CRISPR-Cas system and a self-targeting spacer could potentially also encode an Acr. Rauch et al. (2017) hypothesized that self-targeting spacers would indicate the presence of an inhibiting Acr, which led them to identify four Acrs encoded in prophage regions of *Listeria monocytogenes* that inhibit Cas9. Separately, Watters et al. (2018) and Marino et al. (2018) used a similar approach to identify Acrs in *Moraxella bovoculi* active against type I and type V CRISPR-Cas systems. Given the success in identifying Acrs in prokaryotes harboring self-targeting CRISPR-Cas systems, this mechanism could principally explain the natural appearance of self-targeting spacers.

## Self-Targeting Spacers Underlying Alternative Functions of CRISPR-Cas Systems

We have described how self-targeting spacers can be acquired and how cells can avoid the cytotoxic impact of self-targeting. In some of these cases, self-targeting could reflect an alternative function of the CRISPR-Cas system. Here, we describe different examples in which self-targeting has impacted the host or in which a mechanism has been reported that could impact host behavior, potentially foreshadowing an alternative function. These examples can be divided into four categories: genome evolution, RNA degradation, transcriptional repression, and foreign invaders co-opting self-targeting CRISPR-Cas systems. While conserved examples of CRISPR-Cas systems performing alternative functions have not been described, there has been a steady increase in anecdotal examples that suggest that CRISPR-Cas systems can stray from adaptive immunity, with varying benefits to the host.

### Genome Evolution

One reported outcome of acquiring self-targeting spacers is genome evolution by forcing the host to mutate in order to escape autoimmunity. While this mechanism still reflects active DNA targeting through the standard steps of CRISPR-based immunity and thus may not represent a “true” alternative function, we still consider this an alternative function because of the large-scale change in genomic content that can confer benefits to the host. Specifically, chromosomal targeting can lead to mutations or small deletions in the target gene. These deletions can also be much larger and encompass many surrounding non-targeted genes. While any loss of an essential gene would be lethal, these larger deletions could also provide a fitness advantage by generating new phenotypes or reducing the overall size of the genome, and remodeling of pathogenicity islands could cause a change in bacterial virulence (Vercoe et al., 2013; Westra et al., 2014). Besides triggering active mutations, self-targeting by CRISPR-Cas systems can also select for a small sub-population already lacking the target (Dy et al., 2013; Selle et al., 2015).

Self-targeting by CRISPR-Cas systems can further lead to bacterial or archaeal evolution by disrupting an important gene and forcing the organism to adapt to this change. One important example comes from the bacterium *Pelobacter carbinolicus*. Unlike other members of the *Geobacteraceae* family, *P. carbinolicus* cannot reduce Fe(III) as part of its metabolism (Richter et al., 2007). This phenotype is potentially caused by an existing spacer within the endogenous type I-E CRISPR-Cas system that is complementary to a region within the histidyl-tRNA synthetase gene *hisS*. A lack of histidyl-tRNA synthetase would lead to reduced translation of proteins with multiple closely spaced histidines. The *hisS*-targeting spacer is located opposite of the end of the CRISPR array where new spacers are added, suggesting that the uptake of this spacer did not occur recently. Supporting the active targeting of *hisS*, transforming the self-targeting spacer and the *hisS* gene from *P. carbinolicus* into a genetically tractable strain of the related species *Geobacter sulfurreducens* resulted in few transformants, and these transformants grew poorly. *P. carbinolicus* has also lost or mutated multiple genes with high histidine content that are still present in closely related species, potentially also explaining the loss of Fe(III)-respiration (Aklujkar and Lovley, 2010). It would be interesting to see how the endogenous I-E system is impacting HisS expression without driving lethal autoimmunity, where we expect the mechanism to fall under one of the categories below.

### CRISPR-Cas Induced mRNA Cleavage

Not all CRISPR-Cas systems solely target DNA, wherein RNA targeting could modulate gene expression without inducing cytotoxicity. To-date, type III, type VI, some type I, and some type II CRISPR-Cas systems have been shown to target RNA (Hale et al., 2009, 2012; O’Connell et al., 2014; Samai et al., 2015; Abudayyeh et al., 2016; Li R. et al., 2016; Dugar et al., 2018; Rousseau et al., 2018; Strutt et al., 2018). In the event that RNA but not DNA is targeted, self-targeting spacers would not necessarily result in autoimmunity but instead could degrade mRNA and lead to changes in gene expression.

The type III-B CRISPR-Cas system in *Myxococcus xanthus* is a potential example that degrades mRNA, although this mechanism remains to be fully established (Wallace et al., 2014). As part of the study, the authors performed a transposon screen in a Δ*pilA* strain lacking the type IV pilus required for exopolysaccharide production. They isolated a mutant with a transposon inserted into the CRISPR3 array, which coincided with restored exopolysaccharide production and impaired fruiting body development. Wallace et al. (2014) proposed a mechanism in which the transposon enhanced pre-crRNA processing, leading to crRNA-dependent regulation of exopolysaccharide production and fruiting body development. Other possibilities are that the repertoire of crRNAs includes a portion of the transposon, altering the targeting potential of the array. Given more recent reports of type III-B systems targeting transcriptionally active DNA (Peng et al., 2015; Estrella et al., 2016), other mechanisms may be at work in *M. xanthus* harboring the transposon insertion.

**FIGURE 3:**
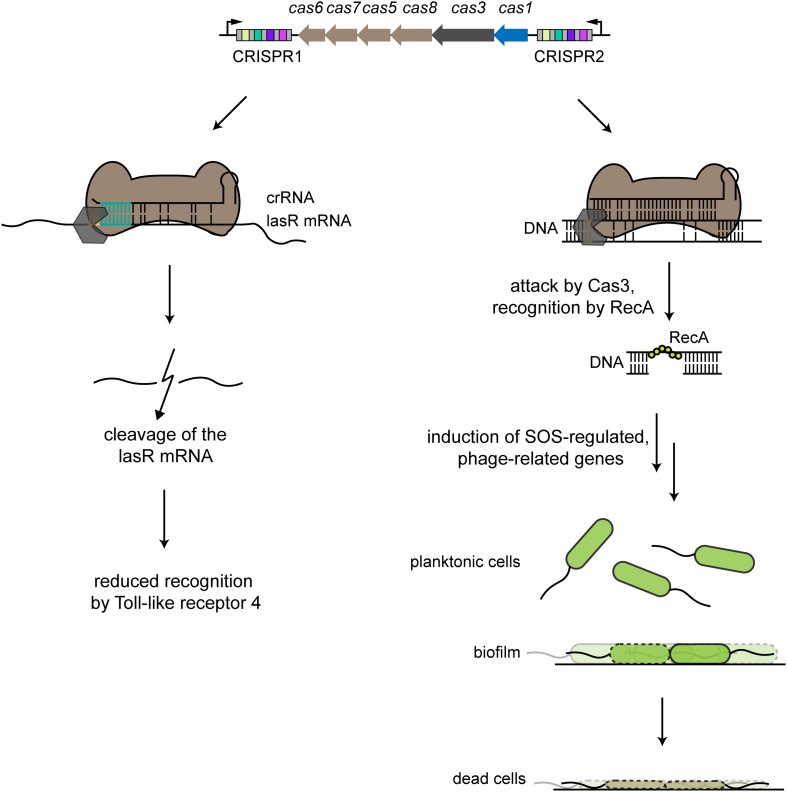
Examples of alternative CRISPR-Cas functions. The type I-F CRISPR-Cas system in *P. aeruginosa* harbors two CRISPR arrays that account for two different alternative functions. The left side shows partial binding between a crRNA derived from the CRISPR1 array and the lasR mRNA, with an indispensable interaction region of 8 nts (turquoise). The Cas effector complex (brown) binds to the target region with an adjacent recognition motif (orange), with some involvement of the Cas3 nuclease. lasR mRNA is then degraded, leading to reduced host recognition by Toll-like receptor 4 during an infection. The right side shows partial binding of a crRNA derived from the CRISPR2 array to a prophage region. Binding by the Cas effector complex recruits Cas3, resulting in nicking of one strand of the target DNA. Recognition by RecA triggers intrinsic processes that lead to induction of SOS-regulated, phage-related genes that lead to cell death of cells specifically forming a biofilm, while planktonic cells are unaffected.

Another alternative function via self-targeting that appears to involve mRNA degradation allows the pathogen *Pseudomonas aeruginosa* to evade immune detection (Figure 3). The type I-F system in *P. aeruginosa* strain UCBPP-PA14 encodes one spacer within its CRISPR1 array that bears partial complementarity to the chromosomally encoded *lasR* gene. LasR is a bacterial quorum sensing regulator whose regulon includes virulence-associated factors presumably detected through Toll-like receptor 4 in mammals. The self-targeting spacer did not lead to any detectable cleavage of the chromosomal DNA but instead appeared to cleave the lasR mRNA. Downregulation of this receptor in turn led to a reduced pro-inflammatory response. The suspected target within the lasR mRNA spans 12 nts, with one internal mismatch and base pairs with the 3′ end of the spacer. Mutational analysis further revealed that disrupting a 5′-GGN-3′ sequence immediately upstream of the lasR target as well as the following 8 base pairs blocked mRNA target degradation (Li R. et al., 2016).

As a brief follow-up to this study, Müller-Esparza and Randau searched for other potentially targeted mRNAs within the *P. aeruginosa* UCBPP-PA14 strain based on potential target sites that include the upstream 5′-GGN-3′ sequence followed by nine complementary nts. They could identify 189 putative targeted mRNAs, suggesting that additional requirements such as mRNA secondary structure are needed for mRNA targeting. Therefore, further studies are necessary to clarify the requirements for mRNA degradation by the type I-F CRISPR-Cas system in this strain of *P. aeruginosa* and the many other organisms encoding these systems (Müller-Esparza and Randau, 2017).

Cas9 is traditionally seen as a DNA-targeting nuclease, yet emerging examples have revealed that some Cas9s can also target RNA (O’Connell et al., 2014; Rousseau et al., 2018; Strutt et al., 2018). Original studies of the Cas9 from *Streptococcus pyogenes* suggested that the effector protein could differentiate between RNA and DNA (Gasiunas et al., 2012), wherein RNA targeting could only be achieved by hybridizing RNA with a PAM-presenting oligonucleotide (PAMmer) (O’Connell et al., 2014; Nelles et al., 2016). Later, it was shown that some Cas9 proteins can cleave RNA even in the absence of a PAMmer. Specifically the Cas9 from the type II-C system in *Neisseria meningitidis* was shown to cleave RNA *in vitro*, while Cas9 from the type II-A system in *S. aureus* and the type II-C system in *C. jejuni* were shown to cleave RNA *in vitro* and *in vivo* (Dugar et al., 2018; Rousseau et al., 2018; Strutt et al., 2018). In all of these cases, RNA targeting did not require a flanking recognition motif. In the example from *C. jejuni*, the naturally occurring spacers were shown to bind and, in some cases, drive Cas9-mediated cleavage of endogenous RNAs. These spacers only exhibited partial complementarity to their targets, and the associated DNA sequences were not flanked by recognized PAMs, preventing genome cleavage. Dugar and coworkers did not explicitly identify a phenotype associated with RNA targeting by the endogenous Cas9 (Dugar et al., 2018), although Strutt et al. (2018) demonstrated that the Cas9 from *S. aureus* could inhibit gene expression through programmable RNA targeting in *E. coli* without leading to cell death. The above mentioned examples show that some DNA targeting systems can also target RNA, with the potential for these same systems to modulate gene expression by RNA degradation in their native hosts.

### CRISPR-Cas Induced DNA Damage Response

The type I-F CRISPR-Cas system in *P. aeruginosa* UCBPP-PA14 performs a distinct alternative function that induces the SOS response, preventing biofilm formation and impairing swarming motility (Zegans et al., 2009; Cady and O’Toole, 2011). A key factor was the presence of a partial match between a spacer within the CRISPR2 array and a sequence present within the lysogenized phage DSM3 (Figure 3). The authors showed that the observed phenotype was dependent not on the presence of the lysogenized phage but rather solely on the target sequence. The presence of the CRISPR-Cas system and the PAM-flanked protospacer led surface-attached cells to undergo cell death, explaining the lack of biofilm formation. The proposed mechanism-of-action involved the recruitment of Cas3 upon binding of the Cascade-crRNA complex to the region of partial complementarity, which recruited RecA and activated the SOS response upon nicking of one DNA strand. Activated RecA also triggered a pathway that led to accumulation of phage-related genes that induced cell death upon surface attachment (Heussler et al., 2015). The ensuing questions are whether this same phenomenon can be found in other biofilm-forming bacteria and whether partial genome targeting can induce other phenotypes.

### Transcriptional Regulation

Beyond RNA targeting, CRISPR-Cas systems have the potential to regulate transcription through partial spacer complementarity or due to the presence of an inactivated nuclease (Sampson et al., 2013, 2019; Ratner et al., 2019). Partial complementarity resulted in regulation of transcription in *Francisella novicida* by so-called scaRNAs (small CRISPR/Cas-associated RNAs). ScaRNAs were encoded close to the CRISPR array associated with the type II CRISPR-Cas system in *F. novicida*. Strictly speaking, the scaRNA-based mechanism is not dependent on a self-targeting spacer but rather on the scaRNA acting as a crRNA. Originally it was hypothesized that the scaRNA targets RNA (Sampson et al., 2013), but later it was shown that the scaRNA hybridizes with the tracrRNA and directs Cas9 to the partially complementary 5′ UTR of its endogenous DNA targets. DNA binding of the target results in transcriptional repression (Ratner et al., 2019; Sampson et al., 2019). In the case of *F. novicida*, targeting with the scaRNA-tracrRNA-Cas9 complex resulted in transcriptional repression of four genes contributing to its virulence by facilitating evasion from immune detection. DNA cleavage by Cas9 is prevented through only partial complementarity of the scaRNA to the target site (Ratner et al., 2019).

Aside from transcriptional repression by DNA binding near promoter regions, another means to regulate transcription is through disruption of the Cas nuclease’s active site. This phenomenon can occur in type I systems that lack the effector protein Cas3 but have an intact Cascade complex (Luo et al., 2015). It is also possible to disrupt the nucleolytic activity of a Cas effector protein by mutating the active site. For example, alanine substitutions in the HNH and RuvC domains in the single effectors Cas9 or Cas12a result in a catalytically dead protein that can bind a target but not cleave it (Bikard et al., 2013; Qi et al., 2013; Leenay et al., 2016). While mutations that solely inactivate cleavage are much less likely than deleterious mutations to the nuclease, either means would result in CRISPR machinery that tightly binds DNA, thereby blocking transcription. Natural examples of catalytically dead CRISPR-Cas systems acting as gene regulators have not been reported, although the ease in disrupting *cas3* in the highly prevalent type I systems would suggest that nature has regularly sampled this alternative function. Screening for CRISPR-Cas systems harboring inactive nucleases and self-targeting spacers or spacers with partial complementarity to the genome might lead to the discovery of further CRISPR-based gene regulatory systems.

### Invaders Co-opting CRISPR-Cas Self-Targeting

There is also evidence of foreign invaders co-opting CRISPR-Cas systems to either promote the spread of MGEs or weaken the host’s adaptive immunity through self-targeting spacers. Recent publications described CRISPR-Cas systems associated with Tn7-like transposons that led to spacer-directed insertion of the transposon (Peters et al., 2017; Klompe et al., 2019; Strecker et al., 2019). The transposon portion of the system generally consists of *tnsB*, *tsnC*, and *tniQ* (a *tnsD* homolog), yet it lacks *tnsD* and *tnsE* normally responsible for recognition of the attachment site (Waddell and Craig, 1988, 1989). Instead, the CRISPR-Cas portion of the system, which lacks nuclease activity and the acquisition machinery, directs transposon insertion up to ∼80 nts downstream of the target site. Because the target site is preserved, an integrated CRISPR transposon would inherently encode a self-targeting spacer (Klompe et al., 2019; Strecker et al., 2019). Nevertheless, the self-targeting spacer appears to be no longer functional due to the lack of multiple transposon insertions at the same target site (Strecker et al., 2019). Acquiring different spacers targeting within the bacteria’s genome would allow the transposon to insert itself elsewhere in the genome, although it is not known how new spacers can be acquired due to the lack of acquisition machinery.

Beyond transposons, phages and viruses also represent types of mobile genetic elements that have co-opted CRISPR-Cas systems for their own purposes. It is reported that some phages or viruses harbor at least parts of CRISPR-Cas systems (Seed et al., 2013; Hooton and Connerton, 2014; Krupovic et al., 2015; Levasseur et al., 2016; Hudaiberdiev et al., 2017; Naser et al., 2017; Dou et al., 2018). One noteworthy example comes from the lysogenic CP8/CP30A phage in *C. jejuni* described earlier. This phage encodes a *cas4*-like gene that is responsible for spacer acquisition within the type II-C CRISPR-Cas system targeting the host’s genome. The authors hypothesized that these self-targeting spacers might provide a benefit for the phage infecting *C. jejuni* and assist in phage-mediated escape from CRISPR attack (Hooton and Connerton, 2014). Phages and viruses could escape from the host immune system by forcing the organism to use its endogenous CRISPR-Cas system for autoimmunity rather than for attacking viral invaders. Furthermore, the organism might mutate or delete its CRISPR-Cas system to prevent cell death and with this also lose the ability to target invading phages or viruses. In total, these examples show that the host and its invaders can utilize CRISPR-Cas systems and their encoded self-targeting spacers for different purposes.

## Conclusion and Future Perspectives

Self-targeting spacers occur surprisingly often in nature, albeit less frequently than spacers matching sequences from known phages, viruses or plasmids. The apparent paradox between the presence of these spacers and their presumed autoimmunity can be resolved in two general ways. These spacers could represent less frequent but important biological “accidents” that compel cells to reduce or eliminate the impact of self-targeting. Alternatively, the cells could be actively using these self-targeting spacers for other purposes that extend beyond adaptive immunity. Both have been reported in the literature, with only a few examples of the latter. However, alternative functions through self-targeting spacers represent an underexplored area of research in CRISPR biology that could yield exciting new insights and tools. Below, we describe multiple opportunities for future research to uncover further instances of alternative functions, advance our understanding of CRISPR biology and evolution, and expand the available toolbox of CRISPR technologies.

One potential focus of future work is on CRISPR-Cas systems encoding multiple self-targeting spacers or on organisms encoding multiple CRISPR-Cas systems. A few examples of bacteria and archaea encoding self-targeting spacers have been reported (Stern et al., 2010) but never explored experimentally. While these examples were categorized as non-effective targeting due to the lack of an apparent PAM, mutated adjacent repeats, extended base pairing with the repeat or lack of some *cas* genes, these sequences could lead to some level of targeting. For instance, CRISPR nucleases are increasingly known to recognize non-canonical PAM sequences (Leenay and Beisel, 2017), and the absence of some *cas* genes could still allow some functions. The accumulation of multiple self-targeting spacers would also suggest a positive selective pressure. One exception could be the disruption of all but Cas1 and Cas2, possibly resulting in acquisition without negative selection against self-targeting spacers. The occurrence of prokaryotes with multiple CRISPR-Cas systems suggests the possibility that some systems could fulfill the canonical CRISPR function as an adaptive immune system and the others might perform alternative functions.

Another potential focus of future work is identifying spacers exhibiting partial complementarity to the host’s genome. As described above, many CRISPR-Cas systems can still bind but not cleave partially complementary targets, resulting in transcriptional repression. Partial complementarity would also allow RNA targeting by some effector proteins, potentially allowing post-transcriptional regulation of endogenous genes. Standard searches for protospacers readily exclude partially matching sequences, owing in part to the difficulty in eliminating false positives. However, regardless of the source of these spacers, partial complementarity with the genome could drive alternative functions. More work is needed to understand what types of mismatches allow different CRISPR-Cas systems to bind but not cleave their targets. This information could then be fed into search algorithms tasked with identifying targets as potential sources of CRISPR-Cas systems moonlighting as gene regulators.

Anti-CRISPR proteins could also provide a potential source for alternative functions. As described above, one strategy to find new anti-CRISPR proteins is to identify organisms with self-targeting spacers (Rauch et al., 2017; Watters et al., 2018). However, the search could be reversed: identifying organisms that harbor both Acrs and CRISPR-Cas systems as potential candidates for identifying systems exhibiting alternative functions. For instance, an encoded Acr that blocks cleavage but not binding activity of the nuclease could convert the immune system into a transcriptional regulator (Pawluk et al., 2018). Discovering new Acrs still remains a major challenge, although further discoveries will enable the search for Acrs tied to alternative functions.

Beyond the discovery of novel instances of functions extending beyond adaptive immunity, interrogating how CRISPR-Cas systems exhibit alternative functions and cope with self-targeting continues to open new biotechnological applications. For instance, the recently discovered CRISPR transposons encoding genome-targeting spacers can serve as powerful tools to insert genes (Klompe et al., 2019; Strecker et al., 2019). Genome-targeting spacers have also been used with classical CRISPR-Cas systems to generate large deletions, representing important capabilities for genome engineering and minimization (Jiang et al., 2013; Oh and van Pijkeren, 2014). As there exist other means by which cells can escape autoimmunity, steps may be necessary to ensure target deletion is the predominant mode of escape. Beyond genome editing, self-targeting with endogenous CRISPR-Cas systems can be part of programmable gene regulation. The endogenous system can be rendered cleavage-deficient while preserving DNA binding activity (Luo et al., 2015). Efforts to interrogate escape from self-targeting have also revealed that gene regulation can be achieved without altering the endogenous system, such as by employing Acrs that inhibit cleavage activity but not DNA binding or by expressing partially complementary spacers. Finally, insights into self-targeting lend to employing endogenous CRISPR-Cas systems as programmable antimicrobials. If the endogenous system is fully active, self-targeting spacers can be used to kill specific bacteria (Bikard et al., 2014; Citorik et al., 2014; Gomaa et al., 2014). If the endogenous system is inhibited by an Acr, relieving expression or activity of these Acrs could unleash lethal autoimmunity, particularly if the endogenous system acquired self-targeting spacers. Further efforts to discover and elucidate new alternative functions could inspire the next generation of CRISPR technologies, emphasizing the need to further investigate the role of self-targeting CRISPR-Cas systems.

## Author Contributions

FW and CB conceived and wrote the manuscript.

## Conflict of Interest

The authors declare that the research was conducted in the absence of any commercial or financial relationships that could be construed as a potential conflict of interest.
